# 2168: Lower rates of influenza infection following 2 dose series of high-dose vaccination in plasma cell disorders: Results of a randomized, double-blind, placebo-controlled study

**DOI:** 10.1017/cts.2017.118

**Published:** 2018-05-10

**Authors:** Andrew Branagan, Eamon Duffy, Terri Parker, Stuart Seropian, Connor Foster, Lin Zhang, Rakesh Verma, Geliang Gan, Daniel Zelterman, Debra Brandt, Jeremy Kortmansky, David Witt, Madhav Dhodapkar

**Affiliations:** Yale School of Medicine, New Haven, CT, USA

## Abstract

OBJECTIVES/SPECIFIC AIMS: (1) Evaluate safety of a novel influenza vaccination strategy in patients with plasma cell disorders. (2) Measure laboratory-confirmed influenza infection rates following a novel influenza vaccination strategy in patients with plasma cell disorders. (3) Evaluate clinical correlates of response following a novel influenza vaccination strategy in patients with plasma cell disorders. METHODS/STUDY POPULATION: We conducted a double-blind, randomized study over the 2015–16 flu season, comparing 2 doses of Fluzone^®^ High-Dose influenza vaccination (separated by 30 d) to the current standard of care influenza vaccination. Patients were allocated to the experimental arm in 2:1 ratio compared with standard of care arm. Standard of care influenza vaccination was considered single age-based vaccination (standard dose for those <65 y and high dosefor those ≥65 y) and patients in this arm received a saline placebo injection at 30 days to assist in blinding. Eligibility criteria allowed any patient with a PCD and no contraindication to trivalent inactivated influenza vaccine. The primary endpoint was laboratory-confirmed flu infection rate. Protocol-driven surveillance screened patients for flu-like illnesses and performed laboratory testing for influenza until the end of the flu season in May 2016. Secondary endpoints include HAI titer serologic response rates, clinical correlates of protection from influenza infection, and exploratory studies of cell-mediated immunity through characterization of T cell subpopulations, cytokine profiles, and flu-specific T-cell responsiveness. RESULTS/ANTICIPATED RESULTS: In total, 122 plasma cell disorder patients were enrolled (97 with disease requiring therapy and 25 with asymptomatic gammopathy). Of those 48 patients received a single standard of care influenza vaccination and 74 patients received 2 doses of Fluzone^®^ high-dose vaccine. Median age was 67 years (range 42–90). This 2-dose vaccination strategy was safely tolerated in all patients with no grade 2 adverse events attributed to vaccine. With close clinical follow-up, only 4% of patients receiving 2 vaccine doses developed laboratory confirmed influenza Versus 8.3% of those receiving single vaccine. When compared to the expected CDC influenza infection rate of 10%–15%, 1 sample, 2-tailed binomial testing revealed patients receiving 2 vaccines experienced a significantly lower rate of infection than the expected rate (*p*<0.05) whereas those receiving single vaccine showed no significant difference (*p*=0.38). DISCUSSION/SIGNIFICANCE OF IMPACT: This randomized study demonstrates that the 2 dose strategy of Fluzone^®^ high-dose influenza vaccine is safely tolerated in patients with plasma cell disorders and associated with significantly less than expected laboratory-confirmed influenza infections. The results suggest that this novel vaccination strategy may have a clinical benefit in reducing influenza infections in plasma cell disorder patients and thus may have practice changing implications. Final analyses of serologic responses, clinical correlates of response, and cell-mediated immune correlates may provide valuable insights into in vivo “immune-competence” in patients with plasma cell disorders.

